# *Anemarrhena asphodeloides* Non-Steroidal Saponin Components Alter the Pharmacokinetic Profile of Its Steroidal Saponins in Rat

**DOI:** 10.3390/molecules200711777

**Published:** 2015-06-26

**Authors:** Zhishu Tang, Guolong Li, Jie Yang, Jinao Duan, Dawei Qian, Jianming Guo, Zhenhua Zhu, Zhongxing Song

**Affiliations:** 1 Shaanxi Collaborative Innovation Center of Chinese Medicinal Resource Industrialization, Shaanxi University of Chinese Medicine, Xianyang 712046, China; E-Mails: weercog@126.com (G.L.); makikoesumi@hotmail.com (J.Y.); dja@njutcm.edu.cn (J.D.); qiandwnj@126.com (D.Q.); szx74816@sina.com (Z.S.); 2 Jiangsu Collaborative Innovation Center of Chinese Medicinal Resources Industrialization, and National and Local Collaborative Engineering Center of Chinese Medicinal Resources Industrialization and Formulae Innovative Medicine, Nanjing University of Chinese Medicine, Nanjing 210023, China; E-Mails: njuguo@njutcm.edu.cn (J.G.); 04040416@163.com (Z.Z.)

**Keywords:** *A. asphodeloides* extract, steroidal saponins, non-steroidal saponins, UPLC-TQ/MS, pharmacokinetics influence

## Abstract

A rapid, selective and sensitive UPLC-MS/MS assay was established to determine the plasma concentrations of four steroidal saponins. Sprague-Dawley rats were allocated to four groups which were orally administered *Anemarrhena asphodeloides* extracts (ASE), ASE combined with macromolecular fraction (ASE-MF), ASE combined with small molecule fraction (ASE-SF) and ASE combined with small molecule and macromolecular fraction (ASE-SF-MF) containing approximately the same dose of ASE. At different time points, the concentration of timosaponin BII, anemarsaponin BIII, timosaponin AIII and timosaponin E1 in rat plasma were determined and main pharmacokinetic parameters including C_max_, T_max_, *T*_1/2_, AUC were calculated using the DAS 3.2 software package. The statistical analysis was performed using the Student’s *t*-test with *p < 0.05* as the level of significance. MF had no effect on the pharmacokinetic behaviors and parameters of four steroidal saponins. It was found that C_max_ and AUC of four steroidal saponins in group ASE-SF and ASE-SF-MF, were significantly increased compared with those in group ASE. These results indicate that SF in *A.* *asphodeloides* extracts could increase the absorption and improve the bioavailability of the steroidal saponins.

## 1. Introduction

*Anemarrhena asphodeloides*, the dried rhizome of *A. asphodeloides* Bunge (Fam. Liliaceae), is mainly distributed in China, Mongolia and other eastern Asian countries. *A. asphodeloides* has been commonly used in traditional medicine in China, Japan, and Korea for thousands of years [1]. In clinical applications of Traditional Chinese Medicine (TCM), *A. asphodeloides* is able to teat febrile diseases, high fever and thirst, lung heat with dry cough, diabetes due to internal heat and constipation [2]. Pharmacological studies revealed that *A. asphodeloides* also possesses anti-microbial activity, decreases the blood glucose level, inhibits platelet aggregation, inhibits carcinomas, decreases radiation injuries and has anti-dementia activity [3,4,5,6].

**Figure 1:**
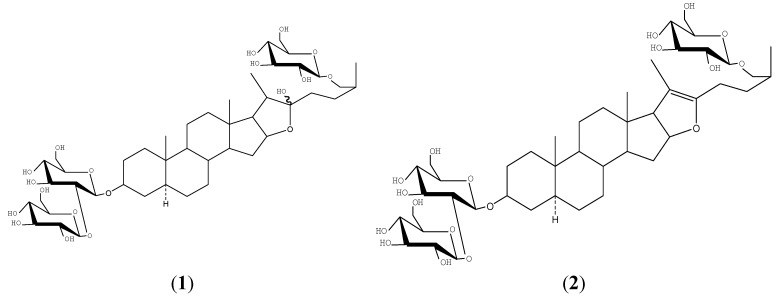

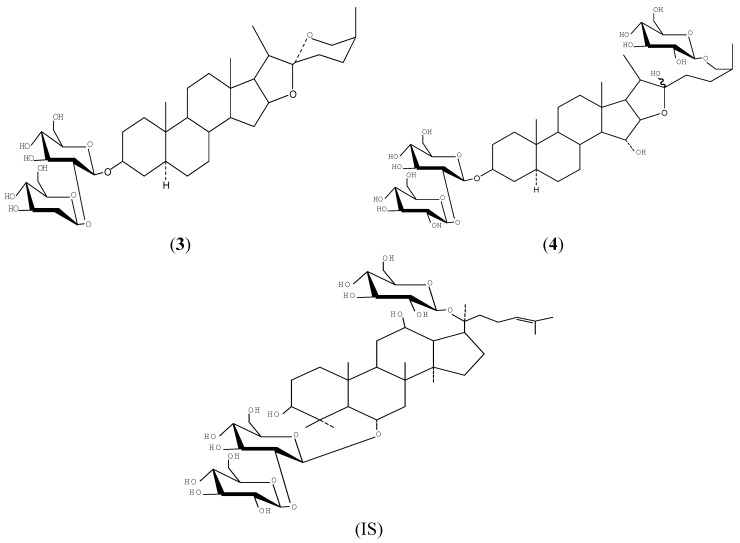
Chemical structures of timosaponinBII (**1**), anemarsaponin BIII (**2**), timosaponin AIII (**3**), timosaponin E1 (**4**) and ginsenoside Re (IS).

As the decoction of the entire rhizome extract is taken orally, the pharmacological activity observed in humans is attributed to the entire rhizome extract, which includes steroidal saponins, flavonoids, pigments, polysaccharides, *etc.* The main active components of *A. asphodeloides* are steroidal saponins, with extremely diverse structures such as timosaponin BII (**1**), anemarsaponin BIII (**2**), timosaponin AIII (**3**) and timosaponin E1 (**4**) (Figure 1), which have been shown to improve senile dementia and have anti-blood coagulation, anti-oxidant, anti-tumor, anti-osteoporosis, anti-inflammation, and blood sugar and blood pressure lowering effects [7,8,9,10,11,12,13]. However, non-steroidal saponin small molecule ingredients such as flavonoids [14,15], organic acids [16,17], amino acids, nucleosides, oligosaccharides and a non-steroidal saponin macromolecule fraction comprising pigments and polysaccharides exist in *A. asphodeloides* [18]. Whether these non-steroidal saponins have pharmacological effects or can influence the absorption of the steroidal saponins ingredients and thus change their bioavailability in the gastrointestinal tract is still unclear.

Several analytical methods are reported for the determination of steroidal saponins in *A. asphodeloides*, including thin-layer chromatography (TLC), gas chromatography (GC) and high-performance liquid chromatography (HPLC) with different detectors [19,20,21]. However, these methods have some limitations including long analysis times and/or low sensitivity and thus are not suitable for the determination of steroidal saponins in biological fluids after administration of *A. asphodeloides* extract. In this study, we hypothesized that the non-steroidal saponin ingredients in *A. asphodeloides* might influence the pharmacokinetics of the active components in the steroidal saponin fraction of *A. asphodeloides*. Therefore, a rapid, sensitive and selective LC-MS/MS method was developed to determine simultaneously four steroidal saponin components in rat plasma and applied to demonstrate the pharmacokinetic influences after comparing the pharmacokinetics of compounds **1**–**4** in the steroidal saponins fraction from *A. asphodeloides* when combined with different non-steroidal saponin fractions. The results are expected to be very helpful for evaluating the effect of non-steroidal saponin ingredients and guiding changes to the dosage form in clinical applications of this herb.

## 2. Results and Discussion

### 2.1. Method Development

#### 2.1.1. Optimization of Mass Spectrometry Conditions

In order to optimize the MS conditions, both positive and negative scan modes were evaluated and the negative mode was selected due to its higher sensitivity, as thee response observed in the negative ionization mode was higher than that in positive ionization mode. MRM mode was used to monitor both quasimolecular and fragment ions, which made the method more specific. ESI source temperature, capillary and cone voltage, flow rate of desolvation gas and cone gas were optimized to obtain the best signal-to-noise ratio of protonated molecules of the four analytes.

#### 2.1.2. Optimization of Chromatography Conditions

To achieve a better peak shape and a shorter running time for simultaneous analysis of the four steroidal saponin compounds, optimization of the mobile phase was conducted. It was recommended that the analysis of furostanol saponins such as timosaponin BII by HPLC-MS be performed using aqueous acetonitrile as mobile phase but not methanol due to the interconversion of the C-22 hydroxy and C-22 methoxy forms [22], hence acetonitrile was chosen as the organic phase. Moreover, the use of 0.1% formic acid in the water phase could improve the ionization efficiency of the analytes and decrease the response intensity of the endogenous matrix, so acetonitrile-0.1% formic acid with gradient elution was applied and selected as the mobile phase with the most favorable retention time and low background noise. Under the developed chromatographic conditions for simultaneous determination of the four compounds, all analytes were eluted rapidly within 4.2 min.

#### 2.1.3. Optimization of Extraction Conditions

In order to obtain a higher recovery of the analytes and IS and no endogenous interference, three types of reagents (*n*-butanol, acetonitrile, and ethyl acetate) were tried for precipitation of protein in rat plasma. It was demonstrated that acetonitrile produced the best extraction efficiency for all the analytes and IS.

### 2.2. Method Validation

#### 2.2.1. Specificity

**Figure 2:**
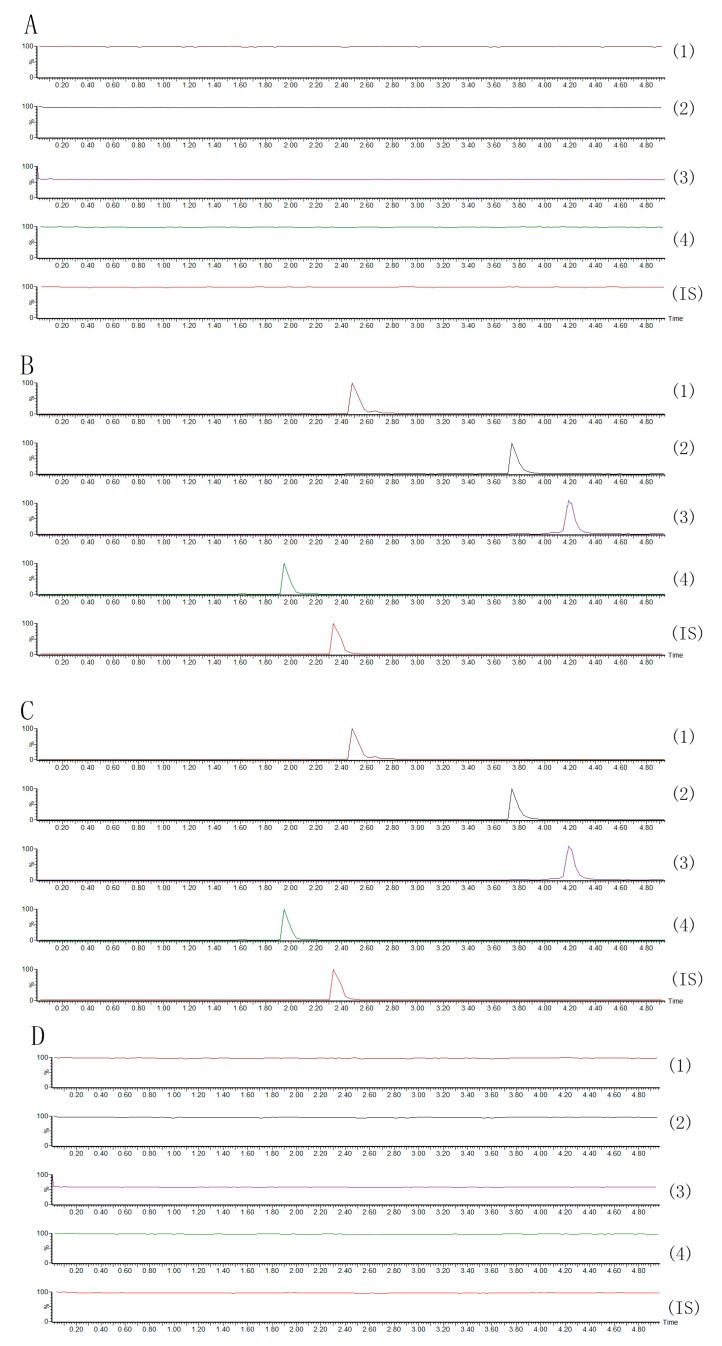
Representatives extract ion MRM chromatograms of compounds **1**–**4** and ginsenoside Re (IS): (**A**) blank plasma; (**B**) blank plasma spiked with each compound; (**C**) plasma sample at 45 min after oral ASE extract; (**D**) blank.

All the analytes and internal standard could be detected on MRM spectrograms without any significant interference (Figure 2).

No endogenous peaks and carryover were observed in the representative chromatograph of blank plasma sample at the retention times of the analytes and IS.

#### 2.2.2. Linearity and Lower Limits of Quantification (LLOQ)

The calibration curves of four compounds exhibited good linearity with correlation coefficients (R^2^) within the range from 0.9919 to 0.9983. The LLOQS were suitable for quantitative detection of compounds in the pharmacokinetic studies. Linear ranges, LLOQs, LLODs and correlation coefficients are shown in Table 1.

**Table 1:** The regression equations, liner range, LLOQs and LODs of the four compounds.

Analytes	Liner Regression Equations	*R^2^*	Range (ng/mL)	LOQ (ng/mL)	LOD (ng/mL)
Timosaponin BII	*Y* = 0.0027*x* + 0.1944	0.9971	5.08–650.00	5.08	1.89
Anemarsaponin BIII	*Y* = 0.0012*x* + 0.0632	0.9919	4.84–620.00	4.84	1.68
Timosaponin AIII	*Y* = 0.0017*x* + 0.1218	0.9983	3.79–485.00	3.79	1.42
Timosaponin E1	*Y* = 0.0018*x* + 0.0983	0.9941	4.14–530.00	4.14	1.29

#### 2.2.3. Precision and Accuracy

The results of the intra- and inter-day precision and accuracy of all the analytes in three QC samples are summarized in Table 2. The intra- and inter-day precisions ranged 4.5%–11.6% and 6.9%–11.2%, respectively. The accuracy derived from QC samples was between 92.7%–106.8% for each QC level of the four analytes. The results indicated that the precision and accuracy values were within the acceptable range.

**Table 2:** The accuracy and intra- and inter- day precision of four analytes in rat plasma (*n* = 6).

Analytes	Concentration ng/mL	Intra-Day		Inter-Day	
		Accuracy (%)	Precision (RSD, %)	Accuracy (%)	Precision (RSD, %)
Timosaponin BII	5.20	86.7	8.8	94.4	10.4
	52.00	97.0	6.4	99.4	8.3
	520.00	92.8	8.5	106.2	11.2
Anemarsaponin BIII	4.96	102.8	8.2	101.2	9.8
	49.60	97.4	11.6	100.0	9.6
	496.00	92.7	6.4	101.6	11.1
Timosaponin AIII	3.88	94.8	11.3	100.9	10.8
	38.80	97.7	9.6	105.5	11.0
	388.00	94.0	9.3	100.2	11.2
Timosaponin E1	4.24	101.0	4.5	104.2	6.9
	42.40	100.6	9.6	106.8	9.2
	424.00	103.3	4.5	96.5	10.2

#### 2.2.4. Extraction Recovery and Matrix Effect

The mean recoveries of all analytes at different concentrations are shown in Table 3. The mean recovery of the analytes was within 73.9%–89.3%. The extraction recovery of the IS was 78.2% ± 9.3%. The matrix effect of blank plasma of all analytes was found to be within the acceptable range; all values were more than 80.7% (Table 3). The matrix effect of IS was 85.4% ± 6.2%. Thus it was indicated that the plasma matrix effect was negligible for the assay.

**Table 3:** Recovery and Matrix effects for four analytes in rat plasma (*n* = 5).

Analytes	Concentration (ng/mL)	Recovery		Matrix Effects	
		Mean (%)	RSD (%)	Mean (%)	RSD(%)
Timosaponin BII	5.20	83.7	6.5	101.3	9.8
	52.00	81.3	8.2	99.9	5.9
	520.00	79.5	9.5	97.6	7.0
Anemarsaponin BIII	4.96	81.7	6.5	99.7	5.1
	49.60	76.1	6.4	80.7	8.5
	496.00	77.8	9.2	96.2	6.1
Timosaponin AIII	3.88	89.3	4.8	92.4	4.7
	38.80	80.7	10.4	95.5	10.3
	388.00	83.5	5.0	89.7	6.2
Timosaponin E1	4.24	88.0	4.3	96.4	6.3
	42.40	73.9	10.1	86.2	10.7
	424.00	77.0	11.7	89.4	8.7

#### 2.2.5. Stability

Stability of the four analytes during the sample storing and processing procedures was fully evaluated by analysis of QC samples. The results are shown in Table 4. The results demonstrated that these analytes in rat plasma were all stable for the auto-sampler for 24 h (4 °C), at −80 °C for 1 month and freeze–thaw cycles with accuracy in range from 83.3% to 103.2%.

**Table 4:** Stability for four analytes in rat plasma (*n* = 6).

Analytes	Concentration (ng/mL)	Auto-Sampler for 24 h		at −80 °C for 1 Month		Freeze–Thaw Cycles	
		Mean (%)	RSD (%)	Mean (%)	RSD (%)	Mean (%)	RSD (%)
Timosaponin BII	5.20	95.3	6.1	94.7	11.8	98.0	12.2
	52.00	98.1	4.0	98.2	3.9	95.5	8.1
	520.00	88.3	5.3	95.4	8.2	91.4	9.5
Anemarsaponin BIII	4.96	97.0	9.7	99.0	4.9	83.3	6.3
	49.60	94.9	6.6	94.3	7.0	93.6	8.2
	496.00	94.9	8.7	91.5	9.8	88.1	7.5
Timosaponin AIII	3.88	100.9	12.6	100.4	10.9	102.2	11.3
	38.80	97.0	12.2	101.7	10.8	96.2	10.7
	388.00	96.4	12.2	99.0	10.3	102.8	6.4
Timosaponin E1	4.24	98.0	3.8	96.8	7.5	103.2	8.8
	42.40	102.3	5.8	93.5	7.9	95.6	9.4
	424.00	99.3	6.1	95.8	10.8	89.8	11.1

### 2.3. Pharmacokinetics Study

**Figure 3:**
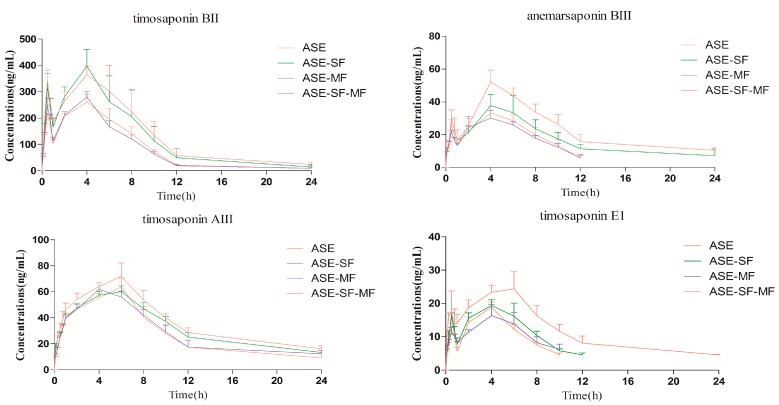
Mean plasma concentration-time curves of steroidal saponins.

The developed and validated method was applied to the pharmacokinetic evaluation of the four steroidal saponins **1**–**4** after oral administration of administration of different fractions to rats (Figure 3). The assay was proved to be sensitive enough for the determination of these analytes in rat plasma.

The pharmacokinetic parameters including maximum plasma concentration (C_max_), time to reach the maximum concentrations (T*_max_*), half-time (*T*_1/2_), area under concentration–time curve (AUC_0–t_) calculated by non-compartment model are displayed in Table 5.

**Table 5:** Pharmacokinetics parameters of four steroidal saponins after an oral administration (*n* = 6).

Analytes	Parameters	Group ASE	Group ASE-SF	Group ASE-MF	Group ASE-SF-MF
Timosaponin BII	C_max_ (ng/mL)	271.22 ± 22.48	415.22 ± 41.79 *	287.22 ± 11.91	400.20 ± 39.57 *
	T_max_ (h)	3.75 ± 1.78	5.00 ± 1.67	3.41 ± 1.43	3.17 ± 2.21
	*T*_1/2_ (h)	3.21 ± 1.17	3.63 ± 1.28	3.34 ± 1.19	5.18 ± 1.88 *
	AUC_0–t_ (ng·h/mL)	2040.97 ± 145.87	3087.78 ± 627.88 *	1983.08 ± 104.24	3314.05 ± 575.12 *
Anemarsaponin BIII	C_max_ (ng/mL)	34.48 ± 3.75	43.47 ± 3.58 *	32.30 ± 4.36	53.28 ± 5.06 *
	T_max_ (h)	3.75 ± 1.78	4.66 ± 1.03	4.00 ± 1.26	4.33 ± 0.81
	*T*_1/2_ (h)	2.75 ± 1.14	5.03 ± 2.71	3.02 ± 1.19	7.63 ± 3.37 *
	AUC_0–t_ (ng·h/mL)	257.15 ± 20.39	406.33 ± 64.49 *	242.35 ± 32.01	550.52 ± 75.03 *
Timosaponin AIII	C_max_ (ng/mL)	65.18 ± 4.16	69.83 ± 2.86 *	63.60 ± 3.16	74.01 ± 6.18 *
	T_max_ (h)	5.66 ± 0.81	5.33 ± 1.03	4.33 ± 0.81	5.66 ± 0.81
	*T*_1/2_ (h)	5.31 ± 3.03	8.20 ± 2.74 *	6.95 ± 6.23	8.94 ± 1.24 *
	AUC_0–t_ (ng·h/mL)	644.27 ± 59.05	778.09 ± 36. 81 *	688.00 ± 46.31	889.76 ± 57.20 *
Timosaponin E1	C_max_ (ng/mL)	18.86 ± 1.54	21.26 ± 2.35 *	17.78 ± 2.85	26.65 ± 2.73 *
	T_max_ (h)	4.00	4.33 ± 0.81	4.66 ± 1.03	5.00 ± 1.09
	*T*_1/2_ (h)	2.95 ± 0.40	3.31 ± 0.48	4.39 ± 2.58	8.80 ± 3.83 *
	AUC_0–t_ (ng·h/mL)	115.38 ± 5.35	145.85 ± 8.28 *	113.21 ± 7.43	286.09 ± 36.84 *

Data are expressed as mean ± S.D. (*n* = 6); * Difference from corresponding Group ASE, *p <* 0.05.

#### 2.3.1. *Comparison of the Pharmacokinetic Profile of the Four Steroidal Saponins 1–4 in the Group ASE and Group ASE-SF*

As shown in Figure 3 and Table 5, the pharmacokinetics parameters of the four steroidal saponins show significant differences in C_max_ .There were no significant differences in T_max_ for the four steroidal saponins **1**–**4** in the ASE and ASE-SF groups. The *T*_1/2_ value of timosaponin AIII was significantly increased. Although the *T*_1/2_ of timosaponin BII, anemarsaponin BIII and timosaponin E1 in the ASE group showed no obvious difference in comparison with those in the ASE-SF group, their *T*_1/2_ values were prolonged. It could be inferred that SF could increase the maximum plasma concentration and extend the elimination time of the four steroidal saponins in rat plasma compared with ASE alone. The AUC of the four steroidal saponins were significantly larger in the ASE-SF group than in the ASE group, which showed that SF may increase the bioavailability of the four steroidal saponins.

#### 2.3.2. *Comparison of the Pharmacokinetic Profiles of the Four Steroidal Saponins 1–4 in the Group ASE and Group ASE-MF*

As shown in Table 5, there are no significant differences among all the groups in Cmax, Tmax, *T*_1/2_ and AUC, indicating that MF have no influences on absorption, elimination and bioavailability of four steroidal saponins in rat plasma.

#### 2.3.3. *Comparison of Pharmacokinetic Profile of Four Steroidal Saponins 1–4 in Group ASE and Group ASE-SF-MF*

C_max_, *T*_1/2_ and AUC of the four steroidal saponins, the major active components of *A. asphodeloides* increased significantly in the ASE-SF-MF group. There was no significant difference in T_max_ for the four steroidal saponins **1**–**4**. The result indicated that SF and MF administered simultaneously with ASE could increase the absorption and improve the bioavailability of four steroidal saponins in rat plasma.

### 2.4. Double Peak Phenomenon

The plasma concentration-time curves of timosaponin BII, anemarsaponin BIII and timosaponin E1 after oral administration showed a double peak, which was also reported in [23,24,25]. It is well known that drug absorption is a very complex process that manifests itself through potential interaction with a host of physicochemical and physiological variables. Some factors that may affect the absorption process include presystemic metabolism/efflux, “absorption window” along the gastrointestinal tract, enterohepatic recirculation, variable gastric emptying and drug-drug interactions [26,27]. Therefore, atypical drug absorption profiles such as double-peaks and absorption window-type absorption profiles are often encountered [27].

Timosaponin AIII is the derivative of timosaponin BII, so they share similar steroid cores. In comparison with timosaponin AIII timosaponin BII only contains an extra sugar moiety in addition to a shared disaccharide moiety, however, their pharmacokinetic parameters are remarkably different. Most obviously, the concentration-time curves of timosaponin AIII after oral administration showed a single peak. There is no scientific report about the aglycone of timosaponin BII. As we know, the aglycone of timosaponin AIII is sarsasapogenin. It is a report of a single plasma concentration peak of sarsasapogenin, which is consistent with the pharmacokinetic behavior of its glycoside (timosaponin AIII). It may be inferred that this sugar is responsible for the different pharmacokinetics [28,29].

### 2.5 Influence of SF and MF on the Pharmacokinetic Profile of Steroidal Saponins

Chinese traditional medicine extracts are an effective aggregation of multiple components, and the pharmacokinetics of a single ingredient cannot represent the pharmacokinetics of the whole herbal medicine. The results of this study implied that SF could increase the absorption and improve the bioavailability of four steroidal saponins from *Anemarrhena asphodeloides* and MF did not significantly improve the bioavailability. However, compared to the ASE-SF group, the values of AUC of ASE-SF-MF increased, so it is unclear if MF can be removed from the clinical medicine so as to alleviate the oral medication burden of patients. However, all these hypotheses need further demonstration.

## 3. Experimental Section

### 3.1. Chemicals and Reagents

The reference standard of timosaponin BII (purity > 98%) was purchased from Chengdu Herb Purify Co., Ltd. (Chengdu, China). Anemarsaponin BIII (purity > 98%) and timosaponin AIII (purity > 98%) were obtained from Chengdu Must Bio-technology Co., Ltd. (Chengdu, China). Timosaponin EI (purity > 98%) was purchased from Beijing Biohalfeast Technology Co., Ltd. (Beijing, China). Ginsenoside Re (purity > 98%) was purchased from the Chinese National Institute of Pharmaceutical and Biological Products (Beijing, China). HPLC grade acetonitrile and formic acid were purchased from Merck (Darmstadt, Germany). Deionized water was purified by an EPED water purification system (EPED, Nanjing, China). All other reagents used were of analytical grade. *A. asphodeloides* was collected from Anhui Province. A voucher specimen (Voucher No. HJ20141211) was deposited at the Shaanxi Collaborative Innovation Center of Chinese Medicinal Resource Industrialization (Xianyang, China).

### 3.2. Chromatographic Conditions 

Chromatographic analysis was performed on an Acquity UPLC system (Waters Corp., Milford, MA, USA), consisting of a binary pump solvent management system, an online degasser, and an autosampler. An Acquity UPLC BEH C18 column (100 mm × 2.1 mm, 1.7 µm) was employed and the column temperature was maintained at 35 °C. The mobile phase was composed of A (0.1% formic acid) and B (acetonitrile) using a gradient elution of 20%–25% B at 0–1.0 min, 25%–30% B at 1.0–3.0 min, 30%–90% B at 3.0–3.1 min, 90%–95% B at 3.1–4.0 min, 95%–20% B at 4.0–4.2 min, at a flow rate set at 0.4 mL/min. The autosampler was conditioned at 4 °C and the injection volume was 2 μL.

### 3.3. Mass Spectrometric Conditions

Mass spectrometry analysis was performed using a Xevo TM triple quadrupole mass spectrometer (Waters Crop., Milford, CT, USA) equipped with an electrospray ionization source (ESI). The ESI source was set in negative ionization mode. The parameters in the source were set as follows: capillary voltage 3.0 kV; source temperature 150 °C; desolvation temperature 550 °C; cone gas flow 50 L/h; desolvation gas flow 1000 L/h. Analytes were performed by using multiple-reaction monitoring (MRM) mode. The cone voltage and collision energy were optimized for each analyte and selected values are given in Table 6. All data collected in centroid mode were acquired using Masslynx4.1 software (Waters Crop, Version 4.1, 2010) and post-acquisition quantitative analysis was performed using the TargetLynx program (Waters Crop, 2010).

**Table 6:** Precursor/product ion and parameters or MRM of compounds used in this study.

Analytes	Retention Time (min)	MRM Transitions (precursor → product)	Cone Voltage (V)	Collision Energy (eV)
Timosaponin BII	2.48	919.5→757.4	52	28
Anemarsaponin BIII	3.74	901.5→739.4	68	30
Timosaponin AIII	4.21	739.4 →577.4	66	40
Timosaponin E1	1.95	935.3→773.2	64	34
Ginsenoside Re	2.33	945.6→637.4	54	34

### 3.4. Preparation of Administered Samples

The dried *A. asphodeloides* were chopped into slices before using. The *A. asphodeloides* was immersed in water (1:10, *w*/*v*) and extracted twice by refluxing for 2 h. After filtration, the supernatant was condensed to a certain volume under reduced pressure, and 95% ethanol was added to the water extract filtrates until the concentration of ethanol was 80%. The precipitate was filtered as the macromolecular fraction (MF), the ethanol supernatant was concentrated under reduced pressure to a certain volume under vacuum and then purified by gradient elution with water, 40% ethanol and 70% ethanol from an AB-8 MARO porous resin column and then the water-eluted fraction was combined with the 40% ethanol-eluted fraction as the small molecule fraction (SF) and the 70% ethanol eluted fraction was the *A. asphodeloides* saponins extract (ASE). All of the residues were lyophilized and the resulting dry powder was stored at 4 °C before usage. The contents of four steroidal saponins in ASE dry powder were measured quantitatively by the external standard method using the same chromatography conditions as described above. The contents of timosaponin BII, anemarsaponin BIII, timosaponin AIII and timosaponin E1 in ASE were 429.12, 89.81, 59.13 and 41.25 mg/g, respectively.

### 3.5. Preparation of Standard Solution and Quality Control (QC) Samples

Stock solutions were separately prepared by dissolving the four accurately weighed standard reference compounds in a mixture of 50% acetonitrile. Then, the four stock solutions were mixed and diluted with 50% acetonitrile to prepare a final mixed standard solution containing 650.00 ng/mL of timosaponin BII, 620.00 ng/mL of anemarsaponin BIII, 485.00 ng/mL of timosaponin AIII, 530.00 ng/mL of timosaponin E1. A series of working solutions were freshly prepared by diluting mixed standard solution with 50% acetonitrile to the appropriate concentration. The internal standard solution of ginsenoside Re was prepared to the concentration of 920 ng/mL in 50% acetonitrile. For the validation of the method, three concentrations of standard solution containing timosaponin BII (5.20, 52.00 and 520 ng/mL), anemarsaponin BIII (4.96, 49.60 and 496.00 ng/mL), timosaponin AIII (3.88, 38.8 and 388 ng/mL), timosaponin E1 (4.24, 42.40 and 424.00 ng/mL) were used for preparing the QC plasma samples.

### 3.6. Preparation of Plasma Samples

Frozen plasma samples were unfrozen at room temperature and treated as follows: to each 200 µL plasma sample, 20 µL of IS (920 ng/mL ginsenoside Re) working solution and 600 µL of acetonitrile were added. After vortexing for 2 min and centrifugation at 13,000 rpm at 4 °C for 10 min the supernatant was transferred into a new tube and evaporated to dryness in a rotary evaporator at 39 °C and the residue was reconstituted in 100 µL of 0.1% formic acid–acetonitrile (50:50, *v*/*v*), vortexed for 2 min and centrifuged at 13,000 rpm at 4 °C for 10 min. The supernatant was transferred to an autosampler vial and an aliquot of 2 μL was injected onto the UPLC-MS/MS system for analysis.

### 3.7. Method Validation

#### 3.7.1. Assay Specificity 

The specificity of the method was evaluated by comparing the chromatograms of six different batches of blank plasma samples, plasma samples spiked with the timosaponin BII, anemarsaponin BIII, timosaponin AIII, timosaponin E1 and IS, and plasma samples obtained from rats administered ASE.

#### 3.7.2. Linearity and lower limits of quantification (LLOQ)

The calibration curves were determined by plotting the peak area ratio (*Y*) of analytes to IS *versus* the nominal concentration (*x*) of analytes with weighted (1/*x*^2^) least square linear regression. The lower limit of quantitation (LLOQ) of the assay was defined as the lowest concentration on the standard curve that can be quantitated with accuracy within 20% bias of the nominal concentration and RSD.

#### 3.7.3. Precision and accuracy 

The intra-day and inter-day precision and accuracy were determined by quantifying three concentration levels of QC samples (six samples for each concentration level) on the same day and on three consecutive validation days, respectively. The precision was expressed as the relative standard deviation (RSD), and the accuracy by (mean measured concentration/spiked concentration) × 100%.

#### 3.7.4. Recovery and matrix effect

The extraction recoveries of analytes at three QC levels were determined by comparing the peak area of each analyte extracted from plasma samples with that of post-extraction spiked plasma blank. The matrix effect was evaluated by comparing the peak areas of the analytes obtained from six plasma samples with the analytes spiked after extraction, at three concentration levels, to those from the neat standard solutions at the same concentrations. The extraction recovery and matrix effect of IS were also evaluated using the same procedure.

#### 3.7.5. Stability

The stability experiments were measured by analyzing replicates (*n* = 6) of three QC samples during the sample storing and processing procedures. For all stability experiments, freshly prepared stability testing QC samples were evaluated by using freshly prepared standard curve for the measurement. The post-preparation stability was tested by determined of the extracted QC samples stored in the auto-sampler (4 °C) for 24 h. The freeze and thaw stability were determined after three freeze-thaw cycles (−80 °C to room temperature). Long-term stability in rat plasma stored at −80 °C was studied for a period of one month.

### 3.8. Pharmacokinetic Study

Male Sprague-Dawley rats (250–280 g) were obtained from the Shanghai Slac Laboratory Animal Co., Ltd. (Shanghai, China). All animals were kept in an environmentally controlled breeding room (temperature: 20–25 °C, humidity: 55%–65%) for 1 week before the experiments started. Animal welfare and experimental procedures were strictly in accordance with the ethical norms of the Nanjing University of Chinese Medicine. All rats were fasted for 12 h with free access to water prior to the experiments, twenty-four rats were divided into four groups and then were given a single dose of ASE, ASE-SF, AS-MF, AS-M-SF. A 40 mg/mL ASE (the amount of MF, SF added to group AS-MF, ASE-SF, AS-M-SF were based on the proportion extracted from *A. asphodeloides*) aqueous solution in each group was administered orally at a dose of 400 mg/kg, which contained 171.61, 35.92, 23.62, 16.53 mg/kg of timosaponin BII, anemarsaponin BIII, timosaponin AIII, timosaponin E1, respectively.

About 400 μL blood samples were collected from venipuncture before intragastric gavage and at 0.083, 0.25, 0.5, 1, 1.5, 2, 4, 6, 8, 10, 12, 24 h after a single oral administration. The blood samples were immediately transferred to heparinized tubes and centrifuged at 3000 rpm for 10 min, and the supernatant was transferred into 2.0 mL Eppendorf tubes and stored at −80 °C prior to analysis. Blank plasma was obtained from the rat without oral administration and was used to investigate the assay development and validation.

### 3.9. Statistical Analysis

To calculate the pharmacokinetic parameters of analytes in different group, concentrations–time dada were analyzed by DAS 3.2 software (Mathematical Pharmacology Professional Committee of China, Shanghai, China, 2011). Data were measured as the mean ± standard deviation (S.D.) with triplicate measurements. The identification of significances between different groups was executed with Student’s *t*-test. A *p* value *<* 0.05 was considered statistically significant.

## 4. Conclusions 

In this paper, a rapid, selective and specific LC-MS/MS method for the simultaneous analysis of four components of *A. asphodeloides* in rat plasma in a simple 4.2 min chromatographic run was developed for the first time. The results obtained from this study implied that after combination with different fractions extracted from *A. asphodeloides* extract, the pharmacokinetic behaviors of the four steroidal saponins showed differences their pure forms or from other extracts. The SF had a significant influence on the pharmacokinetic parameters of the steroidal saponins and the bioavailability and absorption rate were the major parameters which were mainly influenced. But for MF, there was no impact on the pharmacokinetic parameters of the four steroidal saponins. The obtained knowledge might contribute to the safety of clinical therapy and provide valuable information for the pharmacokinetic investigation of TCMs.
